# Benchmarking Strategies for Measuring the Quality of Healthcare: Problems and Prospects

**DOI:** 10.1100/2012/606154

**Published:** 2012-05-01

**Authors:** Pietro Giorgio Lovaglio

**Affiliations:** CRISP and Department of Quantitative Methods, University of Bicocca-Milan, V. Sarca 202, 20146 Milan, Italy

## Abstract

Over the last few years, increasing attention has been directed toward the problems inherent to measuring the quality of healthcare and implementing benchmarking strategies. Besides offering accreditation and certification processes, recent approaches measure the performance of healthcare institutions in order to evaluate their effectiveness, defined as the capacity to provide treatment that modifies and improves the patient's state of health. This paper, dealing with hospital effectiveness, focuses on research methods for effectiveness analyses within a strategy comparing different healthcare institutions. The paper, after having introduced readers to the principle debates on benchmarking strategies, which depend on the perspective and type of indicators used, focuses on the methodological problems related to performing consistent benchmarking analyses. Particularly, statistical methods suitable for controlling case-mix, analyzing aggregate data, rare events, and continuous outcomes measured with error are examined. Specific challenges of benchmarking strategies, such as the risk of risk adjustment (case-mix fallacy, underreporting, risk of comparing noncomparable hospitals), selection bias, and possible strategies for the development of consistent benchmarking analyses, are discussed. Finally, to demonstrate the feasibility of the illustrated benchmarking strategies, an application focused on determining regional benchmarks for patient satisfaction (using 2009 Lombardy Region Patient Satisfaction Questionnaire) is proposed.

## 1. Introduction

Over the last few years, increasing attention has been directed toward the problems inherent to measuring the quality of healthcare. Accreditation and certification procedures have acted as stimulating mechanisms for the discovery of skills and technology specifically designed to improve performance. Total Quality Management (TQM) and Continuous Quality Improvement (CQI) are the most widespread and recent approaches to implementing and improving healthcare quality control [1].

Besides offering accreditation and certification processes, recent approaches measure the performance of health structures in order to evaluate National Health Systems. For example, various international Agencies [2–4] measure the performance of health structures in different countries, considering three main dimensions: effectiveness, efficiency, and customer satisfaction.

In this perspective, performance measurement for healthcare providers, structures, or organizations (from here, hospitals) is becoming increasingly important for the improvement of healthcare quality.

However, the debate over which types of performance indicator are the most useful for monitoring healthcare quality remains a question of international concern [5].

In a classic formulation, Donabedian [6] asserted that quality of care includes (i) structure (characteristics of the resources in the healthcare system, including organization and system of care, accessibility of services, licensure, physical attributes, safety and policies procedures, viewed as the capacity to provide high quality care), (ii) process (measures related to evaluating the process of care, including the management of disease, the existence of preventive care such as screening for disease, accuracy of diagnosis, the appropriateness of therapy, complications, and interpersonal aspects of care, such as service, timeliness, and coordination of care across settings and professional disciplines), and (iii) clinical outcomes.

A clinical outcome is defined as the “technical result of a diagnostic procedure or specific treatment episode” [7], “result, often long term, on the state of patient well-being, generated by the delivery of a health service” [8].

Specifically, ongoing attention has been placed on the importance of combining structural aspects (such as governance and the healthcare workforce) with measures of outcomes to assess the quality of care [6, 9]. This consideration was taken into account by the Institute of Medicine, which, in 1990, stated that “quality of care is the degree to which health services for individuals and populations increase the likelihood of desired health outcomes and are consistent with current professional knowledge” [10].

This definition has been widely accepted and has proven to be a robust and useful reference in the formulation of practical approaches to quality assessment and improvement, emphasizing that the process of care increases the probability of desirable outcomes for patient, reducing the probability of undesired outcomes.

This paper deals with hospital effectiveness, defined as the capacity of hospitals to provide treatment that modifies and improves the patient's state of health. Of particular importance in this perspective is the concept of “relative effectiveness” that is, the effectiveness of each specific hospital in modifying the patient's state of health within a strategy comparing different healthcare institutions, in short, effectiveness evaluation in a benchmarking framework [6].

Benchmarking in healthcare is defined as the continual and collaborative discipline of measuring and comparing the results of key work processes with those of the best performers in evaluating organizational performance [11].

Two types of benchmarking can be used to evaluate patient safety and quality performance. Internal benchmarking is used to identify best practices within an organization, to compare best practices within the organization, and to compare current practice over time. Competitive or external benchmarking involves using comparative data between organizations to judge performance and identify improvements that have proven to be successful in other organizations.

Our aim is to discuss the statistical aspects and possible strategies for the development of hospital benchmarking systems.

The paper is structured as follows: the next section introduces readers to the principle debates on benchmarking strategies, which depend on the perspective and type of indicators used.  Section 3 presents statistical methods, while Section 4 explores the methodological problems related to performing consistent benchmarking analyses.  Section 5 describes an application based on patient satisfaction that demonstrates the feasibility of the illustrated benchmarking strategies.  Section 6 offers conclusions.

## 2. Perspective and Type of Indicators

The conceptual definition and assessment of “effectiveness” rests on a conceptual and operational definition of “quality of care”, which is an exceptionally difficult notion to define.

An important contextual issue is the purpose for which a performance indicator is to be used and by whom.

Performance indicators can be used for various objectives: to gain information for policy making or strategy development at a regional or national level, to improve the quality of care of a hospital, monitor performance of healthcare, identify poor performers to protect public safety as well as to provide information to consumers to facilitate the choice of hospital.

In general, the broader the perspective required, the greater the relevance of outcome measures, as they reflect the interplay of a wide variety of factors, some directly related healthcare, others not. Because outcome measures are an indicator of health, they are valid as performance indicators in as much as the quality of health services has an impact on health. As the perspective narrows, to hospitals, to specialties, or indeed to individual doctors, outcome measures become relatively less indicative and process measures relatively more useful.

Process measures have two important advantages over outcome measures. In fact, if differences in outcome are observed, before one can conclude that the difference reflects true variations in the quality of care, alternative explanations need to be considered. In contrast, a process measure lends itself to a straightforward interpretation (e.g., the more people without contra-indications who receive a specific treatment, the better). Second, the necessary remedial action is clearer (use the treatment more often), whereas for an outcome measure (e.g., higher mortality rate) it is not immediately obvious what action needs to be taken.

Despite these limitations, outcome measures have a role in the monitoring of the quality of healthcare that is important per se. To know that death rates from a specific diagnosis vary across hospitals is an essential finding, even if the reasons for the differences cannot be explained through the quality of care. Further, outcome measurement will reflect all aspects of the processes of care, although only a subset is measurable or measured (e.g., technical expertise and medical skill). Such aspects are likely to be important determinants of outcome in some situations and describe not only that a correct procedure is performed, but also the results for the patients.

Another possible reason why outcome indicators are often used in some countries is that available data refer to routine information systems (administrative archives) which regularly record clinical aspects and other dimension useful for case mix adjustment.

In the Italian context, at patient level, the Hospital Discharge Card (HDC) is the only available administrative archive in the health sector. The HDC, introduced in Lombardy in 1975 with the introduction of reimbursement system of the Diagnostic Related Group (DRG), collects clinical information about patient discharge.

In this perspective, the debate on the use of clinical administrative data to furnish useful information on quality assessment remains open.

Many authors have criticized the use of clinical outcomes in the evaluation of quality of the care and, particularly, mortality rates [12, 13]. According to Vincent and colleagues [14], administrative data does not provide a suitably transparent perspective on quality or improvement. Others suggest that limited clinical content may compromise its utility for this purpose, posing serious caveats against drawing definitive conclusions [15, 16].

Despite such concerns, major consensus exists on the use of clinical outcomes from administrative data as a useful screening tool for identifying quality problems and targeting areas in which quality should be investigated in greater depth [4, 16, 17]. Excluding mortality, various clinical outcomes which could indicate malpractice, are widely accepted by private or public Agencies [1–3, 18, 19] which evaluate national health sectors, for example, unscheduled surgical returns to the operating room within 48 hours, discharges against medical advice, death in low mortality DRGs, or failure to rescue (indicating deaths among patients developing specified complications during hospitalization).

### 2.1. Outcome Variability

In order to consider the methodological problems that may limit benchmark strategies, it is necessary to explore the possible causes of variation in an outcome. Four major categories of explanation need to be considered. The first of these is whether observed differences might be due to differences in the type of patient cared for in the different hospitals (e.g., age, gender, comorbidity, severity of disease, etc.).

The importance of this cause of variation is illustrated by studies where differences in crude outcome disappear when the outcomes are adjusted to take account of these confounding factors. To this end, researchers propose risk-adjustment methodologies as proper methods of equitable comparisons for evaluating quality and effectiveness of hospitals [12, 15, 20].

A second cause of variation in outcome (or its risk-adjusted version) is differences in the way data is collected. Differences in the measurement of events of interest (e.g., deaths) or in the population at risk (typically the denominator of an event rate) depending on different inclusion criteria for determining denominators, or when different case-mix data is used to adjust for potential confounding, will lead to apparent differences in outcome.

Thirdly, observed differences may be due to chance. Random variation is influenced both by number of cases included and by the frequency with which the outcome occurs. To this end, a fundamental issue is whether the outcome indicator is likely to have the statistical power to detect differences in quality. Statistical power depends upon how common the occurrence of the outcome is. For some rare events, the limited number of patients experiencing the events limits the power of the study [21].

Finally, differences in outcome may reflect real, although unobservable, differences in quality of care. This may be due to variations in different measurable or less measurable aspects such as the interventions performed or the skill of the medical team.

Hence, as these are different causes of an outcome variation, the conclusion that a variation in outcome is due to a difference in quality of care among hospitals is essentially a diagnosis through exclusion: if variation cannot be explained in terms of previous components (case-mix, data collection, chance), then hospital quality of care (relative effectiveness) becomes a possible explanation.

## 3. Statistical Methods

As described above, if one cannot explain the variation in terms of differences in type of patient, in how data is collected, or in terms of chance, then quality of care becomes a possible explanation. Following the perspective that variations in outcome are due to a difference in quality of care only as diagnosis through exclusion, institutional agencies gather larger data sets from administrative archives and apply risk-adjustment in order to validate quality indicators and to benchmark hospitals.

Administrative archives are less prone to the problem related to how the data is collected, and reduce the possibility that differences in outcome may be due to chance (although this risk increases when analyzing rare outcomes). Usually, the sizes of such databases cover the entire population of hospitalizations, enhancing their statistical power to detect important differences in outcomes.

Therefore, the last exclusion criterion invokes a consistent statistical model allowing comparisons between hospitals, in order to estimate relative effectiveness [22]. To this end, statistical methods for risk-adjustment identify and adjust variations in patient outcomes stemming from differences inpatient characteristics (or risk factors) across hospitals and, therefore, allow fair and accurate interhospital comparisons.

However, the kind of adjustment required for assessing effectiveness is not the same for the various subjects interested in the results. To this regard, it is useful to distinguish between two types of effectiveness. In fact, potential patients (users) and institutional stakeholders (agents) are interested in different types of hospital effectiveness.

Following the approach of Raudenbush and Willms [23], in a comparative setting, the relative effectiveness is usually assessed through a measure of performance *adjusted *for the factors out of the control of the hospital, so the difference between effectiveness simply lies in the kind of adjustment. The authors identify Type A and Type B relative effectiveness: Type A effectiveness deals with users interested in comparing the results they can obtain by enrolling in different hospitals, irrespective of the way such results are yielded; the performance of the hospital adjusted for the features of its users is evaluated. Type B effectiveness deals with Stakeholders interested in assessing the “production process” in order to evaluate the ability of the hospitals to exploit the available resources; in this case, the performance of the hospital is adjusted according to the features of its users, the features of the hospital itself, and the context in which it operates.

In the nineties, numerous authors proposed to estimate the concept of “relative effectiveness” by means of multilevel or hierarchical models [24, 25]. In fact, when the behaviour of individuals within organizations is studied, the data have a nested structure. Individuals/patients constitute the sampling units at the first and lowest level of the nested hierarchy. Organizations/hospitals constitute the sampling units at the second level.

Several recent statistical papers deal with risk-adjusted comparisons, related to the mortality or morbidity outcomes, by means of Multilevel models, in order to take into account different case-mixes of patients (for a review, see Goldstein and Leyland [26] and Rice and Leyland [27]).

One of the most attractive features of multilevel models is the production of useful results in healthcare effectiveness by linking individual (patient) and organizational (hospital) characteristics (covariates). Multilevel models overcome small sample problems by appropriately pooling information across organizations, introducing some correction or *shrinkage*, and providing a statistical framework that quantifies and explains variability in outcomes through the investigation of patient/hospital level covariates [27].

Quality indicators are typically calculated and disseminated at hospital level, dividing the number of events (in-hospital death or adverse event as a clinical error which results in disability, death, or prolonged hospital stays) by the number of discharged patients at risk.

However, at the patient/individual level, the event of interest is typically a dichotomous variable and the Multilevel model version for this kind of outcome is the Logistic Multilevel Model (LMM, [25]).

For patient *i* nested in hospital *j*, let *π*_*ij*_ be the probability of occurrence of a dichotomous adverse event *Y*_*ij*_, where *Y*_*ij*_ is Bernoulli distributed with expected value *E*(*Y*_*ij*_) = *P*(*Y*_*ij*_ = 1) = *π*_*ij*_. Instead of *π*_*ij*_, the LMM specifies, as dependent outcome, its logistic transformation (*η*_*ij*_ = log⁡⁡(*π*_*ij*_/1 − *π*_*ij*_)) as a function of possible covariates, where log is the logarithmic transformation and (*π*_*ij*_/1 − *π*_*ij*_) the ratio of the probability that the adverse event occurs to the probability that it does not is called the odds of the modelled event.

The LMM without patients and hospital covariates (intercept-only LMM) assumes that *η*_*ij*_ depends only on the particular hospital charging patient *i*, specified by *γ*_0*j*_ a nominal variable designating the *j*th hospital; the hospital effect is assumed to be random, meaning that hospitals are assumed randomly sampled from a large population of hospitals. Equations (1) and (2) define the intercept-only LMM:

(1) $$\begin{array}{c} \eta_{ij}=\gamma_{0j}, \end{array}$$

(2) $$\begin{array}{c} \gamma_{0j}=\gamma_{00}+u_{0j},\;u_{0j}\sim N\left(0,\;\sigma_{0}^{2}\right), \end{array}$$

where *γ*_0*j*_ is the intercept (effect) for the *j*th hospital which can be decomposed in *γ*_00_ representing the average probability of adverse events (in the logit metric) across hospitals and *u*_0*j*_, a specific effect capturing the difference between the probability of adverse event for hospital *j* and the average probability of adverse event across hospitals. These random effects are assumed to be independent and normally distributed with zero mean and variance *σ*_0_^2^, which describes the variability of hospitals' effects. The intercept-only model constitutes a benchmark value of the degree of misfit of the model and can be used to compare models involving different covariates at different levels. Further, this model allows decomposing the total variance of the outcome into different variance components for each hierarchical level. Specifically, the Intraclass Correlation Coefficient (ICC), defined as the ratio between the variability among hospitals *σ*_0_^2^ and total variability (*σ*_0_^2^  plus the variability among patients within the hospitals, *σ*_*e*_^2^) captures the proportion of total variability of a given risk factor that is due to systematic variation between hospitals. Nevertheless, in the case of a dichotomous outcome *Y*_*ij*_, the usual first level residuals *e*_*ij*_, and hence their variance *σ*_*e*_^2^, are not in the model (1). This occurs since the outcome variance *π*_*ij*_/(1 − *π*_*ij*_) being part of the specification of the error distribution depends on the mean *π*_*ij*_ and thus does not have to be estimated separately.

However, approximating the variability of the first level with the variance of the standard logistic distribution (*π* ^2^/3) and summing this variance with the variability of the second level (*σ*_0_^2^) allows separating the total variance in two components, giving the intercept-only model ICC = *σ*_0_^2^/(*σ*_0_^2^ + *π* ^2^/3). This measure is used to assess the percentage of outcome heterogeneity existing between the hospitals involved in the analysis.

As the second step, the probability (in the logic metric *η*_*ij*_) of an adverse event occurrence for patients can be a function of patients' characteristics (case-mix), other than the hospital effect. Hence (1) can be extended assuming that *η*_*ij*_ depends on *P*  (*p* = 1,…, *P*) patient covariates (*x*_*pij*_)

(3) $$\begin{array}{c} \eta_{ij}=\gamma_{0j}+\overset{P}{\underset{p=1}{\sum }}\gamma_{pj}x_{pij}, \end{array}$$

(4) $$\begin{array}{c} \gamma_{0j}=\gamma_{00}+u_{0j}, \end{array}$$

(5) $$\begin{array}{c} \gamma_{pj}=\gamma_{p0}+u_{pj}, \end{array}$$

where *γ*_*pj*_ is the slope (regression coefficient) of the *p*th person characteristic in hospital *j* which is allowed to randomly vary across hospitals (e.g., the effect of length of stay on adverse event occurrence varies among hospitals). In the formulation (4), the specific effect for the *j*th hospital on the outcome (*u*_0*j*_) is adjusted for the effects of the *P* person-level characteristics (*x*_*ijp*_). In (5) *γ*_*p*0_ represent the average slope across hospitals and *u*_*pj*_ the specific effect of hospital *j* to the average slope (random effect). However, in effectiveness analyses, slope parameters (*γ*_*pj*_) are assumed to be fixed (putting *u*_*pj*_ = 0 in (5) for  *p* = 1,…, *P*), whereas only the intercept *u*_0*j*_ is allowed to randomly vary across hospitals. Such models, in which the regression slopes are assumed fixed, are denoted as *variance component *models.

In the model composed by (3)-(4) and (5) with  *u*_*pj*_ = 0, the *u*_0*j*_ reflects the relative effectiveness of the *j*th hospital, depurated only by individual case-mix characteristics, and thus potentially depending on different hospital characteristics (Type A effectiveness).

For Type B effectiveness, one can move to the next step, accounting for variation in intercept parameters across hospitals by adding *Q*  (*q* = 1,…, *Q*) hospital variables *z*_*qj*_ to level 2 equations. Hence, (4)-(5) become

(6) $$\begin{array}{c} \gamma_{0j}=\gamma_{00}+\overset{Q}{\underset{q=1}{\sum }}\gamma_{0q}z_{qj}+u_{0j}, \end{array}$$

(7) $$\begin{array}{c} \gamma_{pj}=\gamma_{p0}+\overset{Q}{\underset{q=1}{\sum }}\gamma_{pq}z_{qj}, \end{array}$$

in which slope parameters (*γ*_*pj*_) referring to (3) are specified as nonrandom covariates across hospitals, but possibly varying depending on characteristics of hospital *j*(*z*_*qj*_).

Methodologically, this step is justified when in the model (3)-(4) the intercepts *u*_0*j*_ do significantly vary across hospitals (by investigating the associated residual ICC), once the patients' characteristics are controlled for.

The compact form of (3)-(6)-(7) is

(8) $$\begin{array}{cc} \eta_{ij} & =\gamma_{00}+\overset{P}{\underset{p=1}{\sum }}\gamma_{p0}x_{pij}+\overset{Q}{\underset{q=1}{\sum }}\gamma_{0q}z_{qj}\\ & \;+\overset{P}{\underset{p=1}{\sum }}\;\overset{Q}{\underset{q=1}{\sum }}\gamma_{pq}x_{pij}z_{qj}+u_{0j}, \end{array}$$

where the double sum in (8) captures possible cross-level interactions between covariates at different levels (e.g., *γ*_*pq*_ exhibits that, for hospital *j*, the effect of length of stay (*x*_*pij*_) on adverse event occurrence (*η*_*ij*_) may depend on the specialisation level *z*_*qj*_ of the hospital).

In model (8), the parameters *u*_0*j*_, called level 2 residuals, specify the relative effectiveness of the hospital *j* (Type B effectiveness): they show the specific “managerial” contribution of the *j*th hospital to the risk of adverse event, depurated by overall risk (*γ*_00_), individual case-mix (∑_*p*_ *γ*_*p*0_ *z*_*pij*_), structural/process characteristics of the hospitals (∑_*q*_ *γ*_0*q*_ *z*_*qj*_), and their interactions (∑_*p*_∑_*q*_ *γ*_*pq*_ *x*_*pij*_ *z*_*qj*_). To make this interpretation clear, (8) can be rewritten by isolating the *u*_0*j*_ in the right term of expression (8): the effectiveness parameter *u*_0*j*_ is thus a hospital unexplained deviation of the actual outcome (*η*_*ij*_) from the expected outcome (*γ*_00_ + ∑_*p*_ *γ*_*p*0_ *x*_*pij*_ + ∑_*q*_ *γ*_0*q*_ *z*_*qj*_ + ∑_*p*_∑_*q*_ *γ*_*pq*_ *x*_*pij*_ *z*_*qj*_). The expected outcome is the outcome predicted by the model based on the available hospital and patient-level covariates. For patient *i* of hospital *j*, the difference between actual and expected outcome has a hospital-level component *u*_0*j*_ (the effectiveness). Notice that, since the expected outcome depends on the covariates, the meaning of effectiveness depends on how the model adjusts for the covariates (Type A or Type B).

One method of estimating *u*_0*j*_ is to use the empirical Bayes (EB) residual estimator [24]. The EB estimator can be interpreted as the difference between the “average” we actually observe for a hospital (average of the actual outcome for a hospital) and the “average” that is expected for the hospital after controlling for the individual and hospital factors that influence the average (average of the expected outcome for a hospital). Hence, adjusting for both individual and hospital level sources of variation, the EB residual is that part of the evaluation of the variable at hand (adverse event occurrence) that we believe to be due to management practices. The exponential value of the estimated hospital-specific random effect *u*_0*j*_ is the odds ratio (OR): the odds of experimenting an adverse event at the *j*th hospital divided by the odds of an average hospital, after controlling for the individual and hospital factors. Patients who are treated at hospitals with positive random effects (OR > 1.0) have greater odds of adverse event than patients who are treated at an average hospital, whereas patients who are treated at hospitals with negative random effects (OR < 1.0) have lower odds of adverse event than patients who are treated at an average hospital.

However, since the residuals are affected by the sampling variability and other sources of error, the corresponding ranking has a degree of uncertainty. Such uncertainty is difficult to represent, since it involves multiple comparisons. If Hospital A's risk-adjusted outcomes are significantly better than those of Hospital B's, then we are more confident that Hospital A offers high quality of care, but we cannot assume that Hospital A is actually better than Hospital B. Therefore, several authors [4, 8, 27] suggest avoiding hospital rankings based on their risk-adjusted outcomes, but to place hospitals into a limited number of groups, based on statistical criteria. In a conservative approach, the usual procedure is to build 95% pairwise confidence intervals (CI) of level 2 residuals, or their exponentiated values, and situate hospitals into three groups: effective (problematic) hospitals are those with CIs entirely under (over) the risk-adjusted mean (e.g., regional) of warning event, whereas CIs that cross the risk-adjusted mean define the intermediate group. Further, the effectiveness of two hospitals is statistically different whether the 95% pairwise ICs of *u*_0*j*_ do not overlap.

### 3.1. Case-Mix Adjustment

Typically, appropriate adjustment instruments must control for the principal diagnosis within a Diagnostic-Related Group-(DRG) (categorization of each hospitalization based on the average resources used to treat patients), contain demographics as proxies for preexisting physiological reserve (e.g., gender, age, marital status, socioeconomic status), and measure the number and severity of comorbidities [28].

Comorbidities, or coexisting diseases, are obtained by DRG and principal-secondary diagnoses, whereas comorbidity severity is measured with different strategies: among others, (i) aggregating comorbidities reflecting different conditions leading to hospitalization [29], (ii) aggregating DRG reflecting admission gravity (disease staging, [4, 30]). For example, disease staging maps from the list of comorbid diagnoses to a severity scale that ranges from 1 to 4 where stage one is the least severely ill and stage four is death. In absence of institutional software measuring severity, possible alternatives contained in Hospital Discharge Cards data are length of stay, admission type (planned/urgent), hospitalization type (surgical/other), DRG, and DRG weight, a numeric value assigned to each discharge based on the average resources consumed to treat patients in that DRG.

In this end, risk-adjustment methods that use only administrative data appear to be a viable alternative to widely accepted severity adjustment methods when additional clinical data (medical chart, laboratory values, etc.) required by existing severity adjustment strategies are not available [31].

### 3.2. Decomposing Total Variance

Various approaches have been proposed to examine the proportion of explained variance and to indicate how well the outcome is predicted in a multilevel model. A straightforward approach consists of examining the residual error variances and residual ICC in a sequence of models.

However, in an LLM, if we start with an intercept-only model, and then estimate a second model where we add a number of covariates (the linear predictor in (3)), we normally expect the variance components to become smaller. However, in logistic regression the first level residual variance is again *π* ^2^/3. These implicit scale changes make it impossible to compare regression coefficients across models, or to investigate how variance components change [25]. One possible solution is a multilevel extension of a method proposed by McKelvey and Zavoina [32] that is based on the explained variance of a latent outcome in a generalized linear model. In this formulation, for a specific model with *m* covariates, the variance of *η*_*ij*_ is decomposed into the first level residual variance, which is fixed to *π* ^2^/3, the second-level intercept variance *σ*_0_^2^  and the variance *σ*_*F*_^2^ of the linear predictor (obtained by calculating the variability of the predictions arising from the fixed part of the model). The variance of the linear predictor is the systematic variance in the model, whereas the other two variances are the residual errors at the two levels. In this specification, we can rescale the variance estimates *σ*_0_^2^ and *π* ^2^/3 of a specified model with *m* covariates by an appropriate scale correction factor, that rescales the model to the same underlying scale as the intercept-only model. Let *σ* ^2^ = *σ*_0_^2^ + *π* ^2^/3 denote the total variance of the intercept-only model, and *σ*_*m*_^2^ = *σ*_0_^2^ + *π* ^2^/3 + *σ*_*F*_^2^ for the “model *m*” including *m* first-level covariates. Applying the scale correction factor *σ* ^2^/*σ*_*m*_^2^ to the variance components of model *m*, the corrected variance components can be used for assessing ICC and the amount of variance explained at the two levels.

### 3.3. Aggregate Data and Rare Events

Often dichotomous data may be available at higher levels than the patient level (e.g., aggregated adverse events occurring in the *k*th Specialty belonging to hospital *j*). In that case, the individual dichotomous outcome *Y*_*ij*_ becomes a proportion or an event rate, defined as the number of events divided by the total person of experience (*π*_*kj*_). Specifically, *π*_*kj*_ is the ratio between *Y*_*kj*_, the counts of adverse events occurring in *k*th Specialty of the *j*th hospital (stratum *kj*), and *n*_*kj*_, the size of the population at risk in stratum *kj*. Conditional on the covariates, *Y*_*kj*_ is assumed to have a binomial error distribution, with expected value *π*_*kj*_ and variance  *π*_*kj*_(1 − *π*_*kj*_)/*n*_*kj*_, where *n*_*kj*_ is the number of trials or the population at risk (e.g., discharged patients) in stratum *kj*.

In this case, with aggregate data, we can continue to use LMM. Here, first level refers to Specialty *k*, instead of patient *i*. In each stratum *kj*, we have a number of patients who may or may not experiment the adverse event. For each patient *i* in stratum *kj*, the probability of a warning event is the same, and the proportion of respondents in the *k*th Specialty of the *j*th hospital is *π*_*kj*_, which is the dependent outcome to be modelled. This formulation does not model individual probability and does not use individual-level covariates. However, in the presence of individual dichotomous data (*Y*_*ikj*_ for patient *i* in the stratum *kj*), we could have a model where each individual's probability varies with individual-level covariates in a three-level model.

With aggregate data, another possible way to model proportions is to use regression count models. Count data is increasingly common in clinical research [33]. Examples include the number of adverse events occurring during a follow-up period or the number of hospitalizations. Poisson Regression (PR, [34]) is the simplest regression model for count data and assumes that each observed count *Y*_*kj*_ is drawn from a Poisson distribution with the conditional mean *μ*_*kj*_ on a given vector *x*_*kj*_ for stratum *kj*. If *Y*_*kj*_ is assumed to be drawn from a Poisson distribution, the mixed Poisson regression is useful if researchers are interested in whether the (logarithm of) expected rates  (*μ*_*kj*_/*n*_*kj*_), which are incidence densities, varied across Specialty and hospital characteristics or not. Here, *n*_*kj*_ may denote both the size of the population at risk in stratum *kj* or the size of the time interval over which the events are counted varies. Indicating *η*_*kj*_ = log⁡(*μ*_*kj*_/*n*_*kj*_), once having substituted index *i* with index *k*, (8) identifies the Poisson Multilevel Model. It involves, as the dependent variable, an event rate, such as the ratio of clinical errors resulting in patient death to the total discharges in the *k*th Specialties belonging to hospital *j* or the number of clinical errors resulting in patient death per charge period. The random error *u*_0*j*_ continues to represent the specific managerial contribution of hospital *j* to the rate of clinical errors, once Specialties characteristics (case-mix) and hospital structural characteristics are taken into account.

The main feature of the Poisson model is that the expected value of the random variable *Y*_*kj*_ for stratum *kj* is equal to its variance. However, its assumption of equi-dispersion, resulting in an underestimation of the outcome variability, is too restrictive for many empirical applications. In practice, the variance of observed count data usually exceeds the mean (overdispersion), due to the unobserved heterogeneity and/or when modelling rare events. In this situation, one classic cause of over-dispersion is the presence of the excess of zeroes in the analyzed outcome distribution (e.g., when many hospitals are not responsible for adverse events). Ignoring over-dispersion seriously compromises the goodness of fit of the model, which also leads to an overestimation of the statistical significance of the explicative variables.

In this perspective, as described in the previous sections, a fundamental issue for statistical models is whether the outcome indicator is likely to have the statistical power to detect differences in quality. In the presence of a rare event, the small number of patients experiencing said event limits the power of the study (at a given significance level) and one cannot conclude that some hospitals are better than the rest, or that a specific hospital with low performance (high complication rate) is worse, as these differences might have arisen by chance.

When the data show over-dispersion and excess of zeros (rare events) compared to the expected number under the Poisson distribution, other count models, such as the Negative Binomial Regression model (NBR, [34]) and Zero-Inflated regression models, appear to be more flexible. NBR is able to model count data with over-dispersion, because NBR is the extension of PR with a more liberal variance assumption, modelled by means of a dispersion parameter. Instead, Zero-Inflated regression models address the issue of excess zeroes in their own right, explicitly modelling the production of zero counts. Specifically, it is assumed that there are two processes that produce the data: some of the zeros are part of the event count and are assumed to follow a Poisson model (or a negative binomial). Other zeros are part of the event taking place or not, a binary process modelled in a binomial model (logistic equation). These zeros are not part of the count; they are structural zeros, indicating that the event *never *takes place.

Thus, for count data with the evidence of over-dispersion and when over-dispersion results from a high frequency of zero counts (rare events), several modelling strategies give satisfactory fitting measures.

### 3.4. Continuous Outcomes

The rationale underlying the specification of (8) can be generalized to the case in which the outcome variable is assumed to be continuous (or is a scale in which the responses to a large number of questions are summated to one score) with a normal error distribution. However, two main differences arise. Firstly, in a Linear Multilevel Model [24], instead of modelling the logit of *Y*_*ij*_, we directly model *Y*_*ij*_ and, secondly, the model now involves the level 1 residuals *e*_*ij*_ (assumed to have a normal distribution with zero mean and variance *σ*_*e*_^2^ and to be independent from the level 2 residuals *u*_0*j*_). The parameter can be estimated by the full or restricted maximum likelihood method [24].

In the intercept-only model, the ICC  ( = *σ*_0_^2^/(*σ*_0_^2^ + *σ*_*e*_^2^)) indicates the proportion of the variance explained by the grouping structure in the population. Since, with additional covariates, all residual variance components become smaller, at each step, we can decide which regression coefficients or variances to keep based on the significance tests, the change in the deviance, and changes in the variance components (residual ICC).

When the response variable does not have a normal distribution, the parameter estimates produced by the maximum likelihood method are still consistent and asymptotically unbiased, meaning that they tend to get closer to the true population values as the sample size becomes larger. However, the asymptotic standard errors (variance-covariance matrix of the estimated regression coefficients) are incorrect, and they cannot be trusted to produce accurate significance tests or confidence intervals for fixed effects [24, page 60]. One available correction method to produce robust standard errors is the so-called Huber/White or sandwich estimator [35], where variances of the estimated regression coefficients are obtained by empirical residuals of the model (robust standard errors). This makes inference less dependent on the assumption of normality. 

Further, when the problem involves violations of assumptions and the aim is to establish bias-corrected estimates and valid confidence intervals for variance components, a viable alternative to asymptotic estimation methods is the bootstrap [25].

### 3.5. Outcomes Measured with Error

In specific circumstances, effectiveness analyses may be conducted by using quality of life outcomes (or patient satisfaction) which can constitute the basis for assessing different hospitals in a comparative setting. Quality of life indicators refer to the general condition of health of the patient (physical and mental health, functional state, independence in daily living, etc.) and describe the conditions in which services are distributed. 

Although such variables are not directly observable, they can be estimated by analyzing tests administered to patients. Suppose we wish to analyze the data of a given class of *n* independent subjects. Let *ξ* denote the latent outcome (or patient satisfaction). The associated Linear Multilevel Model is

(9) $$\begin{array}{c} \xi_{ij}=\gamma_{00}+u_{0j}+\overset{P}{\underset{p=1}{\sum }}\gamma_{pj}x_{pij}+e_{ij}, \end{array}$$

where *e*_*ij*_, conditioned on variables in the linear predictor and *ξ*, have zero mean and variance *σ*_*e*_^2^ and *u*_0*j*_, conditioned on covariates and *ξ* are independent normal variables with zero mean and variance *σ*_0_^2^. However, *ξ*_*ij*_ is latent and we only observe a fallible measurable version (*Y*_*ij*_^*o*^). In accordance with the Classical Test Theory, which assumes that the observed scores for *K* tests measure the same true latent outcome score, plus an error term, this defines an explicit measurement model for the latent outcome:

(10) $$\begin{array}{c} Y_{ij}^{o}=\xi_{ij}+\delta_{ij},\;\delta_{ij}\;|\;\xi_{ij}\sim N\left(0,\;\sigma_{i}^{2}\right) \end{array}$$

in which the error term *δ*_*ij*_ is normally distributed with zero mean and variance *σ*_*i*_^2^, which varies across subjects (*i* = 1,…, *n*) in the same manner across hospitals. For example, *Y*_*ij*_^*o*^ can be thought as the total score obtained by summing scores for patient *i* in hospital *j* over *K* administered tests or as a composite score, estimated by using one of the known models for continuous latent variables. From (10) we can decompose the variance (Var) of *Y*_*ij*_^*o*^ as the sum of its orthogonal variance components:

(11) $$\begin{array}{c} \mathrm{Var}\left(Y_{ij}^{o}\right)=\mathrm{Var}\left(\xi_{ij}\right)+\sigma_{\delta }^{2}, \end{array}$$

where *σ*_*δ*_^2^ = *N* ^−1^Σ_*i*_ *σ*_*i*_^2^ denotes the average of the individual standard errors of the measurement.

In such circumstance, when the variable measured with errors is the response variable of the model, its measurement error is captured by the model error and there are no consequences on the estimated parameters, but this has serious consequences on variance components. In fact, (11) illustrates that, due to measurement error, the variance of the estimated latent variable overestimates the true latent variable variance.

Therefore, since instead of *ξ*_*ij*_ we observe an error-contaminated estimation *Y*_*ij*_^*o*^, by adding *δ*_*ij*_ to both terms, the model (9) becomes

(12) $$\begin{array}{c} Y_{ij}^{{}^{\circ}}=\xi_{ij}+\delta_{ij}=\gamma_{00}+u_{0j}+\overset{P}{\underset{p=1}{\sum }}\gamma_{pj}x_{pij}+e_{ij}+\delta_{ij} \end{array}$$

in which *σ*_*δ*_^2^ (the variance of measurement errors) enters as an additional random component in the total variance of *Y*_*ij*_^*o*^, thus modifying formulas to obtain ICC.

For the intercept-only model, ICC = *σ*_0_^2^/(*σ*_0_^2^ + *σ*_*e*_^2^ + *σ*_*δ*_^2^), which resulted in an attenuated version of the true ICC, thus underestimating the variability of outcome across hospitals. Hence, when the outcome is measured with error, ICC must be disattenuated (ICC^§^), by subtracting the term *σ*_*δ*_^2^ in the denominator of the attenuated ICC.

To this end, different approaches can be utilized to estimate *σ*_*δ*_^2^ (and thus ICC^§^). These concerns can be addressed within the context of Rasch measurement models [36] providing measures underlying Likert scales with optimal characteristics. The Rasch model directly furnishes individual estimates of *σ*_*i*_^2^ (the standard error of the estimated outcome for person *i*, measured across *K* items), and averaging them provides an estimate of *σ*_*δ*_^2^. 

Another possibility deals with factor analysis (FA). Without loss of generality, let us consider *K* congeneric tests, allowing different error variances for *K* tests and removing the assumption that all tests are based on the same units of measurement. Supposing that the scores of *K* items for n subjects are embedded in the vector of *K* variables **Y** ^o^ = (*Y*_1_^o^,…, *Y*_*K*_^o^)′, and let **Y** ^o^ = ***λ*** *ξ* + **δ** denote a single-factor analysis model for *K* items, where ***λ*** = (*λ*_1_,…, *λ*_*K*_)*'* and **δ** = (*δ*_1_,…, *δ*_*K*_)*'* indicate the vector of partial regression coefficients of *ξ* in the regression of **Y** ^o^ on *ξ*, and the error terms, respectively. In the FA model, *σ*_*δ*_^2^ can be estimated once the reliability of the composite *ρ* = 1 − (*σ*_*δ*_^2^/*σ* ^2^), defined as the ratio of true variance to observed score variance *σ* ^2^, is estimated.

Unlike traditional methods for computing composites as total scores, the use of maximally reliable composite scores [24] minimizes measurement error in the items contributing to each scale, thus increasing the reliability of the computed scale scores. More specifically, let ξ* = ***λ*** ^∗′^Σ^∗−1^ **Y**° denote the factor score estimates for the individuals, where Σ* is the estimated covariance matrix of the observed indicators and λ* the estimated vector of regression coefficients; the reliability of the composite ξ* is estimated as

(13) $$\begin{array}{c} r=\frac{w^{*\prime }\;\left(\Sigma^{*}-\Theta^{*}\right)w^{*}}{\left(w^{*\prime }\Sigma^{*}w^{*}\right)}, \end{array}$$

where w* is the estimated vector of factor score regression weights (w* = ***λ*** ^∗′^Σ^∗−1^) that maximize *r* and Θ* is the diagonal matrix of estimated error terms variances *δ*_*K*_.

Finally, measurement error bias becomes more serious when the model involves a covariate measured with error (e.g., when the outcome at baseline is used as a covariate to estimate performances), causing bias in the estimated parameters. This arises because the measurement error of the outcome at baseline is correlated with *e*_*ij*_ + *δ*_*ij*_ in (12).

## 4. Methodological Problems

As described, the proposed analyses on large administrative archives can be used for benchmarking purposes. Notwithstanding the illustrated advantages, these analyses also present specific challenges, due to the following potential areas for bias.

### 4.1. The Risk of Risk Adjustment

Firstly, risk adjustment can only adjust for factors that can be identified and measured accurately (case-mix fallacy). Consequently, risk adjusted benchmarking, using administrative data, can be hampered by underreporting, that is, the potential endogeneity of the recorded patient-level covariates (outcomes are correlated with the propensity to record information across hospitals) and the potential for nonconsidered covariates (misspecification). For example, if an important severity measure is missing from the database, assuming that the distribution of this unmeasured covariate will vary across hospitals, the variability of adjusted outcomes among hospital may be overestimated [30].

Furthermore, when using administrative archives for adverse events, claims data is problematic in nature, given the limited number of claims generally emerging from administrative sources (underreporting, or lack of close calls or near misses/errors that do not result in injury) and the lack of information on the causes of medical errors causing injury to patients (e.g., processes and systems of care that may be responsible).

Secondly, unmeasured risk factors are not randomly distributed across hospitals, due to clustering of certain types of patients in certain hospitals' practices. Users can easily draw incorrect conclusions, because the hospitals that appear to have the worst outcomes may simply have the most seriously ill patients. To this end, the practice of routinely disseminate risk-adjusted hospital comparisons has been strongly criticized, since an institution's position in rankings strongly depends on the method of risk adjustment used [37].

Third, since differences in the quality of care within hospitals (e.g., DRGs and/or Specialties) may be greater than differences between hospitals, there is no clear evidence of high correlation between how well a hospital performs on one standard of effective care and how well it performs on another. After risk adjustment, the remaining hospitals variability (type B effectiveness) may be imputable to complex factors, typically depending on a reciprocal interaction between patient case mix (pathologies, clinical severity) and the institutional form of the hospital (profit, not-for-profit/public, private, University hospital, etc.). Therefore, the unexplained hospital variability appears to be physiological and not possible to eliminate completely [8, 22, 37]. In this perspective, it has become imperative to evaluate which benchmarks keep the risk of comparing noncomparable hospitals to a minimum.

To this end, some authors [38] propose to use additional factors, which contribute most to variability in patient experience, as supplementary adjustment variables for patient mix or as stratification variables in order to present transparent benchmarking analyses.

### 4.2. Selection Bias

Patient selection bias is a distortion of results due to the way subjects are selected for inclusion in the study population. Patients are not randomly assigned to hospitals. Whereas randomized and controlled trials reduce self-selection bias through randomization by evenly distributing subjects among treatment/hospital, observational studies based on administrative database are nonrandomized and effectiveness results may be confounded by selection bias due to systematic differences in admission practices between (private/public) hospitals or differences in hospital referral patterns. Such selection biases may result in the preferential admission (or exclusion) of patients with different underlying prognoses, independently of the severity of patients' illness.

Estimates of the effects and outcomes can be biased due to a correlation between factors (such as baseline health status) associated with hospital selection and outcomes (endogeneity). In fact, effectiveness random parameters *u*_0*j*_ are assumed as independent and uncorrelated with fixed explicative variables. When this correlation occurs (e.g., this may occur since the patients are selected in hospital), the hypothesis is not valid and the model is not appropriate. Such a correlation can result in erroneous inferences about the magnitude and statistical significance of hospital effects [25]. Assessment of such bias, which limits a suitable relative effectiveness of hospitals [39], would be extremely difficult and would require information about all possible hospital admissions.

A straightforward remedy to endogeneity due to a possible covariate *x*_*p*_ is to add the hospital mean of *x*_*p*_ to the model equation: this makes the patient level covariate *x*_*p*_ uncorrelated with the hospital effects, so valid estimates of the Type A effects can be obtained. In this sense, the bias is shifted to Type B effects by the endogeneity of hospital-level covariates that typically occurs for the omission of relevant covariates at this level.

Furthermore, to control for selection bias in observational data, different statistical techniques can be used for evaluating hospital effectiveness that adjust for observed and unknown differences in the baseline characteristics and prognostic factors of patients across hospitals. Propensity Score (PS), Instrumental Variable (IV), and Sample Selection Models (SSM) are three techniques developed to minimize this potential bias [39, 40].

PS is the individual probability that a patient will receive a particular treatment (i.e., chooses hospital *j*) and is estimated by logistic regression that predicts a patient's choice as a function of covariates, including patients' pretreatment characteristics (sociodemographic, comorbidities, diagnosis, and urgency-related factors). Using PS, potential bias due to hospital choice is minimized if the choice and the outcome being evaluated are conditionally independent given the measured pretreatment characteristics.

Further, in a second stage, ad hoc models (e.g., LMM or multilevel version of count regression models when data are aggregated) are used to estimate relative effectiveness across hospitals in the outcome equation, adjusting for posttreatment characteristics and propensity scores. This can be done by adding PS as additional continuous covariate or by estimating hospitals effectiveness in the outcome equation within propensity scores strata, typically quintiles.

Sample Selection Models (SSMs) attempt to control the bias introduced by unobserved variables in hospital selection, which are also correlated with the outcome of interest. SSMs, widely used in the econometrics literature, are a special case of Instrumental Variable (IV) Models. The concept behind an IV is to identify a variable, the “instrument,” that is associated with a subset of the variables that predict hospital choice but is independent of the patient's baseline characteristics. If a good IV is identified, both measured and unmeasured confounders can be accounted for in the analysis.

Typical instruments include severity of illness, territorial supply of healthcare providers that may or may not offer specific treatments the distance from each patient home to either the nearest hospital that does specific treatments; or the nearest hospital, that may or may not provide specific treatments [41].

SSMs are two-stage methods. Before estimating the outcome equation (second-stage model), the probability that patient *i* has chosen hospital *j* is predicted as an endogenous variable, as a function of observed patient and hospital characteristics, including instrumental variables. Further, all instrumental variables are excluded from the second-stage model.

The residual from the first stage is then added as an explanatory variable to the outcome equation. It captured the unobservable nonrandom component and allowed us to control for selection bias. Instead, IV techniques, contrary to SSMs, use a single equation to estimate the relative effectiveness without estimating the choice equation that is replaced by the presence of instruments in the outcome equation.

## 5. Application

To clarify the potentiality of the presented methods, this section focuses on hospital effectiveness concerning patient satisfaction. In Lombardy, the monitoring of patient satisfaction, mandatory for hospitals, is performed using the Official Customer Satisfaction (OCS) questionnaire of the Lombardy region. It contains 12 items regarding acceptance, healthcare performance, satisfaction with physicians and nurses, accommodation, discharge, and two items asking for an overall judgement of satisfaction. Each item is scored on a seven-point Likert scale ranging from 1 to 7. Scores of 5 and over indicate increasing levels of satisfaction*,* whereas scores of 3 and below indicate dissatisfaction.

Available data, provided by the regional Directorate of Healthcare, refers to all Lombard hospitals in 2009, which between April and November 2009, delivered the OCS questionnaires to a random sample of discharged patients, proportional to their annual number of discharges in 2009.

For the analysis, we select only patients with planned admissions to general hospitals (excluding urgency admissions and specialist hospitals) in order to minimize the risk of patient selection for analysed hospitals. Globally, the sample is composed by 46,096 patients, nested in 64 hospitals (an average of 720 patients per hospital). Exploring the patient covariates embedded in the OCS, patients differ by gender (46% are female), age class (7% < 24 years, 37% in the age class 25–54 and 55% > 54 years), schooling level (5% primary school, 50% middle school, 36% high school, 9% university degree), and nationality (94% are Italian).

Available hospital structural characteristics involve sector (Private/public), typology (University or not), size (in three bed-size categories), and whether the hospital has an emergency unit. Hospital process measures (all measured in 2009 and obtained by Hospital Discharge Cards) involve number of specialties in the hospital (N_Specialties), percentage of beds utilized (% Beds), number of operating rooms utilized (N_OpRoom), total number of hours operating room utilized (Hours_OpRoom), average monthly hours per operating room (Ave_MH_OpRoom), and the case-mix of charged patients during 2009.

The case-mix is measured as the percentage of (surgical and medical) discharges having DRG weight above (High case mix) or below (Low case mix) the regional median DRG weight. In the analyzed sample, 52% are public hospitals, 85% have emergency unit, 8% are University hospitals, and 36% have more than 250 beds (5% < 50 beds).

Analyzing items scores (Table 1) with Confirmative Factor Analysis, we found three orthogonal (Varimax rotation) composites: the first deals with clinical aspects satisfaction (Y1: ClinSAT), the second with general and accommodation aspects of satisfaction (Y2: GenSAT), and the third coincides with the single item dealing with satisfaction on waiting time to be admitted in hospitals (Y3: WaitLists). For the first two, coefficients alphas (*α*_Y1_ = 0.92; *α*_Y2_ = 0.90) and composite reliability (*r*_Y1_ = 0.89; *r*_Y2_ = 0.84) indicate acceptable internal consistency and reliability for the estimated composites.

Despite many patients being very satisfied in many domains (column 3 of Table 1), a multilevel analysis is performed to assess whether there are meaningful differences between hospitals in evaluations of patient satisfaction and whether these differences remain, after controlling for patient and hospital characteristics (hospital effectiveness). Specifically, we specify a Linear Multilevel Model for the composites Y1 and Y2, whereas a Logistic Multilevel Model is used for predicting the probability of being dissatisfied with waiting time, using as dependent outcome Y3d (WaitDISSAT), a dichotomous variable that is equal to 1 when the score on the Waiting time item ≤3 and is equal to 0 otherwise.

The upper part of Table 2 exhibits, for Y1 and Y2, the corrected (disattenuated) ICCs in the intercept-only model and the residual ICCs (the remaining proportion of variability due to hospitals differences, once that covariates are inserted in the models). For Y3d, the Residual ICC is rescaled with the scale correction factor, in order to be comparable to the ICC of the intercept-only model. 

The three patient outcomes appear to be highly influenced by the inclusion in the different hospitals; for continuous outcomes, the disattenuated ICCs (higher than the attenuated versions that equal 8.2% and 10.4% for Y1 and Y2, resp.) demonstrated that a high proportion of the differences in the outcomes is attributable to differences between hospitals. This especially occurs for Y2, meaning that almost 15% of the variance in overall satisfaction (14.8%) is across hospitals. 

To explain these differences, available covariates are used. The lower part of Table 2 exhibits covariates that are significant at least for one outcome. Firstly, individual patient characteristics and other hospital characteristics (such as the chirurgical case-mix, hospital dimension, and presence of emergency unit) are found to be not significant (at the 0.05 significance level).

This highlights that the three patient satisfaction dimensions are not affected by patient characteristics and do not significantly vary among available hospital characteristics.

In contrast, for Clinical Satisfaction (Y1), most of the variation is associated to the difference in the number of specialties (inversely linked with Y1) and of the number of operating rooms (positively linked with Y1) between hospitals, with higher levels of Y1 for university hospitals, demonstrating that clinical satisfaction is higher in specialized university hospitals.

The overall satisfaction (Y2) is higher for private hospitals with high volumes of operating room hours utilized and decreases for hospitals with several specialties and high utilization rates of operating rooms. Observing Y3d, it is of note that the significant covariates for predicting overall satisfaction (Y2) act in exactly the same manner in predicting the dissatisfaction for waiting time, (higher for private hospitals with several specialties) and the high utilization rates for operating rooms.

After checking for hospital characteristics, the residual ICCs become very small, except for Y2 that decreases to 9.5% from 14.8%. Globally, the significant hospital covariates explain 81%, 34%, and 90% of the outcome variability among hospitals for Y1, Y2, and Y3d, respectively.

The remaining hospital differences (residuals) are purported to define effects of management practices (Type B effectiveness) to increase patient satisfaction in the three domains.

Before investigating the obtained rankings, we explore possible covariate endogeneity by means of three generalized linear models which specify, for each outcome, the hospital residuals (*u*_0*j*_) as dependent variable. In these models, the effects of hospital covariates are found to be not significant (at the 0.01 significance level).

The global *F*-tests, referring to the hypothesis that all covariates' coefficients are equal to zero, versus the alternative that at least one does not, are largely not significant (*F*_Y1_ = 0.41, *P*-value = 0.954; *F*_Y2_ = 0.49, *P*-value = 0.913; *F*_Y3d_ = 0.51, *P*-value = 0.987), meaning that no serious endogeneity is found, so valid effectiveness parameters are obtained.

As a last step of the analysis, we check the concordance of three hospitals rankings based on the estimated *u*_0*j*_ (Type B effectiveness). Spearman correlations (*r*) exhibit weak agreement between estimated rankings for all outcomes, showing three independent dimensions. Specifically, the ranking based on overall satisfaction is significantly and positively correlated with the ranking based on clinical satisfaction (*r* = 0.375, *P*-value = 0.002) and with those based on satisfaction with waiting time (*r* = 0.304, *P*-value = 0.014), although of modest strength. Instead, the correlation between the rankings of clinical and waiting time satisfaction is positive, but at the limit of statistical significance (*r* = 0.252, *P*-value = 0.045).

## 6. Conclusion

Using clinical outcomes for quality assessment represents an important approach to documenting the quality of care. Consumers of indicator information (stakeholders, clinicians, and patients) need reliable and valid information for benchmarking, making judgments, and determining priorities, accountability, and quality improvement.

Where health services have effects on outcome, use of outcome measures as performance indicators is appropriate and efforts should be taken to ensure that the benchmarking strategies can be interpreted reliably. However, the conclusion that differences in outcome are due to differences in quality of care will always be tentative and open to the possibility that the apparent association between a given unit and poor outcome is due to the confounding effect of some other factor that has not been measured, measured inadequately, or misspecified.

As the empirical application has shown, estimated hospital rankings must be interpreted in scrupulous detail. Despite such limitations, clinical administrative data is broadly considered as a useful screening tool for identifying quality-related problems and in targeting areas, which potentially require in-depth investigation. The simultaneous monitoring of several outcomes, which indicate malpractice appears to offer a useful strategy in facilitating hospitals and stakeholders in detecting trends and identifying extreme outliers.

Once a benchmark for each performance measure is determined, analyzing data results becomes more meaningful.

However, moving from the evaluation step towards the phase of statistical implications mainly depends on the way in which monitored (e.g., adverse) events are distributed among hospitals. If a large proportion of adverse events are concentrated among relatively few hospitals, the traditional quality control approach targeting error prone, ineffective health structures for specific attention has high potential value. When variation is discovered through continuous monitoring, or when unexpected events suggest performance problems, members of the organization may decide that there is an opportunity for improvement.

The opportunity may involve a process or an outcome that could be changed to better meet customer feedback, needs, or expectations.

In contrast, when ineffective hospitals are more diffusely distributed, targeting specific hospitals may be a less efficient strategy than investigating the clinical processes in the framework of continuous quality improvement with an emphasis on careful examination, rigorous, scientific testing methods, statistical analysis, and the transparent adjustment of clinical processes.

To this end, exhaustive and exclusive measure specifications should be described, including specific definitions of the clinical indicators and standards and identification of the target population and data sources.

Steps can be taken to minimize the possibility of a false conclusion being drawn on the quality of care based on outcome measurement.

Standardising how data is collected can reduce the extent to which differences in measurement can potentially cause observed variation. Including sufficient numbers of patients will reduce the possibility of random variation masking real differences or making spurious differences appear. Development of sophisticated case-mix adjustment systems can reduce the possibility that observed differences are due to differences in the types of patient, developing of an analytical plan with descriptions of the statistical and clinical significance of results to be assessed when comparing groups or comparing a group to a standard. 

As part of the development process, indicator measurement can be made more efficient when incorporated into routine patient care as part of clinicians' and administrators' documentation of required information on patient characteristics and care delivery, already being recorded for clinical purposes (medical record data). This would eliminate duplicative clinical data collection for the purposes of clinical care and quality assessment.

In conclusion, another important topic that affects the evaluation quality of the care in a benchmarking perspective is the institutional condition of the healthcare system and its modifications over time.

For example, the English National Health Service (NHS) has developed from 2002 onwards a new era of hospital market (New Labour). Under this model, competition arises from patient choice, selective contracting of purchasers (primary care trusts) with providers and from competition between different providers (NHS trusts, private providers, independent sector treatment centres, and NHS foundation trusts).

In Italy, since 2001, the healthcare system has moved in the direction of a welfare-mix system, characterized by freedom of choice for the consumer and by the joint-presence of state agents (operating with functional financial autonomy), private profit or nonprofit accredited companies endowed with autonomous decision-making and managerial procedures and by freedom of choice for the consumer.

Hence, the specific question is to evaluate the relation between hospital competition and hospital quality. To this end, some recent econometric studies focusing on NHS find causal effects of hospital competition on care quality. Specifically, they show that competition improves clinical quality (as measured by reduction in hospital mortality rates after myocardial infarction) and also reducing waiting times [42, 43].

In this perspective, other open questions remain crucial: does available evidence-based result support institutional proposals to extend competition? How does competition compares with other policies to increase hospital quality? More applied research is required for these topics.

Overall, the present paper suggests a launching board for discussions with experts in the field of administrative data, risk adjustment, and performance measurement reporting. Clinicians and researchers should actively participate in designing future administrative databases to ensure that they are clinically meaningful and useful for quality measurement, offering regional stakeholders the opportunity to gain a deeper understanding of the problematic areas in clinical risk assessment.

## Figures and Tables

**Table 1:** Item Analysis: missing values, percentage of patients satisfied, and item-component correlation (*n* = 46, 096).

	Missing	% Satisfied	Y1	Y2	Y3
Item description	values	(scores 6+7)	ClinSAT	GenSAT	WaitLists
Nurses' courtesy, attention, availability	370	88.5%	**0.70**	0.04	0.09
Doctors' courtesy, attention, availability	606	89.4%	**0.83**	0.19	0.08
* Satisfaction of the care* provided	1309	89.3%	**0.81**	0.06	0.07
Health status (and discharge) information	609	85.1%	**0.79**	0.06	0.05
Privacy and consent information	635	88.6%	**0.72**	0.11	−0.05
Comfort, bed, food, cleanliness	2150	83.6%	0.12	**0.78**	0.11
Organisation of the process of the care	627	81.6%	0.11	**0.78**	0.02
Recommend hospital (friends or relatives)	1346	85.2%	0.12	**0.73**	−0.03
Overall satisfaction	704	85.3%	0.05	**0.72**	0.01
Waiting time to be admitted to the hospital	1417	75.7%	0.01	−0.02	**0.99**

**Table 2:** ICC and significant hospital characteristics.

	Y1	Y2	Y3d
	ClinSAT	GenSAT	WaitDISSAT
ICC	13.0%^§^	14.8%^§^	12.2%
Residual ICC	2.7%^§^	9.5%^§^	1.2%^#^

Hospital Characteristics	Model coefficients and significance		

Private Hosp	n.s	2.068**	0.0420***
University Hosp	1.729**	n.s	n.s.
% Beds	−0.020*	−0.056**	n.s.
N_ Specialties	−0.079***	−0.281***	−0.0040***
N_OpRoom	0.072***	−0.102*	n.s.
% High medical casemix	3.515*	n.s	n.s.
Hours_OpRoom	n.s	0.001***	n.s.
Ave_MH_OpRoom	n.s	−0.058***	−0.0004*

^§^corrected for measurement error, ^#^rescaled with scale correction factor.

*** P-value < 0.01, **P-value < 0.05, *P-value < 0.10, n.s. = not significant.
